# Occupational and Non-occupational Injuries Can Result in Prolonged Augmentation of Psychiatric Disorders

**DOI:** 10.2188/jea.JE20200374

**Published:** 2022-01-05

**Authors:** Wei-Shan Chin, Shih-Cheng Liao, Shin-Chun Pan, Yue-Liang Leon Guo

**Affiliations:** 1 School of Nursing, College of Medicine, National Taiwan University (NTU) and NTU Hospital, Taipei, Taiwan; 2 Department of Psychiatry, College of Medicine, National Taiwan University (NTU) and NTU Hospital, Taipei, Taiwan; 3 National Institute of Environmental Health Science, National Health Research Institutes, Zhunan, Taiwan; 4 Department of Environment and Occupational Medicine, College of Medicine, National Taiwan University and NTU Hospital, Taipei, Taiwan

**Keywords:** psychiatric disorders, occupational injury, trauma and stress-related disorder, depression

## Abstract

**Background:**

The long-term effects of occupational injury (OI) on psychiatric diseases are unclear. This study assessed and compared the effects of OI, no injury (control), and non-OI (NOI) on the development of psychiatric diseases.

**Methods:**

We used Taiwan’s National Health Insurance Research Database to investigate the incidence of psychiatric disorders in OI, NOI, and control groups. The subjects were aged 20–50 years, actively employed in 2000, and did not have history of injury or psychiatric disorders. All subjects were followed from 2000 and were classified into OI, NOI, and control groups according to occurrence of target injury later on. Individuals in each group were matched by age, sex, insurance premium before the index date, and year of the index date. Psychiatric disease-free days were compared among the groups using survival analysis and Cox regression.

**Results:**

We included a total of 12,528 patients for final analysis, with 4,176 in each group. Compared with the control group, the OI group had an increased occurrence of trauma and stress-related disorder, depressive disorders, anxiety disorders, and alcohol and other substance dependence. These increases were similar to those in the NOI group. Elevated cumulative incidence rate of any psychiatric disorders was observed among those with OI or NOI up to 10 years after injury.

**Conclusion:**

We confirmed that OI and NOI induced psychiatric disorders. These findings highlight the need for workers’ compensation mechanisms to consider long-term psychological care among injured workers.

## INTRODUCTION

The Global Burden of Disease study by World Health Organization estimated that the total disability-adjusted life years (DALYs) caused by occupational injury (OI) decreased by 0.6% between 2006 and 2016, with more than 22 million DALYs being observed in 2016.^1^ In addition to experiencing physical impairment, 5.2–7.5% of injured workers have been reported to develop either posttraumatic stress disorder (PTSD)/partial PTSD or major depression within 1 year after OI.^2^^,^^3^ Furthermore, the findings of previous surveys conducted in Taiwan and the United States have shown that the risk of psychiatric disorders was higher in patients with OI than in those without injury.^4^^,^^5^ However, these two studies had follow-up periods of no longer than 2 years.

Several studies have suggested that traumatic events or natural disasters may result in long-term psychiatric disorders.^6^^–^^9^ However, little is known about long-term psychiatric consequences occurring after OI. In Taiwan, Chin and her colleagues discovered that, in contrast to general belief, time did not attenuate psychiatric conditions up to 6 years after OI.^10^ Prior surveys have recruited only workers who scored high in psychological symptoms in the first stage of the questionnaire survey and then diagnosed workers’ mental disorders through telephone interviews. This could lead to potential bias in the selection of subjects for interview and underestimate the results. In addition, because of the lack of a comparison with a control group, one cannot clearly determine the actual effect of OI on psychiatric consequences.

To address the aforementioned gap in knowledge, we used a nationally representative cohort (Taiwan National Health Insurance [NHI] database) to determine whether psychiatric diseases were elevated after occupational injury, as compared to uninjured workers.

## METHODS

### Data sources

The Taiwan NHI program is a universal single-payer health insurance system that was established by the Taiwanese government in 1995 to deliver universal health care coverage for all citizens. The NHI program has covered more than 99% of the 23 million residents of Taiwan.^11^^,^^12^ In 1997, the National Health Research Institutes (NHRI) established the National Health Insurance Research Database (NHIRD) for academic research. The NHIRD includes information of all clinical visits (outpatient, inpatient, and emergency medical service), prescription details, and diagnostic codes based on International Classification of Diseases, Ninth Revision, Clinical Modification (ICD-9-CM) codes.^13^

The Longitudinal Health Insurance Database 2005 (LHID2005) is a subset of the NHIRD, and it is a random sample of 1 million people (4.3% of all beneficiaries) who were alive and insured by the NHI in 2005. All claims data of these 1 million individuals were collected to constitute the LHID2005. The NHRI confirmed that the LHID2005 is a representative cohort of the Taiwanese population and that there is no significant difference in the distribution of age, sex, and insurance premium between individuals in the LHID2005 and those in the original NHIRD.^13^ In this study, we used the LHID2005 for the period from 1997 to 2013.

### Ethical approval

This study was approved by the Institutional Review Board of National Taiwan University Hospital (approval number: 201807017W) and complied with the principles outlined in the Declaration of Helsinki.

### Identification of the study cohort

We used a retrospective matched-cohort design in the present study. All eligible participants aged 20–50 years in 2000 were enrolled from the LHID2005 and were employed at the time they experienced the first injury or received upper respiratory infection (URI) diagnosis from 2000 to 2013. A column named “Gave kind” in the NHIRD was used to indicate whether this medical record was occupational-related. Among the four possible Gave kind codes, 1, 2, 3, and 4 represented “OI,” “occupational disease,” “NOI,” and “nonoccupational disease,” respectively.^13^ First, we identified 9,945 subjects who had been hospitalized due to OI (Gave kind code 1) from 2000 until the end of 2013. This group was classified as the OI group. Second, subjects who had been hospitalized due to injury (ICD-9-CM codes 800.xx to 999.xx) from 2000 until the end of 2013 and did not belong to the OI group were defined as the NOI group. Third, to recruit adequate controls who had ever sought medical attention, subjects who had been treated for acute respiratory infection (ICD-9-CM codes 460.xx to 466.xx) between 2000 and 2013 and did not receive any injury diagnosis were categorized into the control group.

In the aforementioned groups, we defined the date of the very first diagnosis for injury or URI as the index date. We excluded those subjects who had a psychiatric history (ICD-9-CM codes 290.xx to 319.xx) before the index date, those who had an injury history before 2000, and those who were unemployed (insurance unite type code “51, 52, 61, 62, 62S, 62T” / those were insured by their relatives ID RELATION code “3, 4, 5, 6, 7, 8, 9, U”) in 2000.

Minimizing the effect of socioeconomical status and injury severity in mental health, after identifying potential subjects in each group, the final subjects for analysis were selected by matching the age in 2000, sex, insurance premium before the index date, and year of index date. In OI and NOI groups, we recorded individuals’ hospitalization period immediately after injury to be as the severity of the injury. Insured premium is directly related to salary income, making it a good indicator of socioeconomic status. The period of hospitalization is related to the severity of injury, and was included in the model.

**Figure 1.**
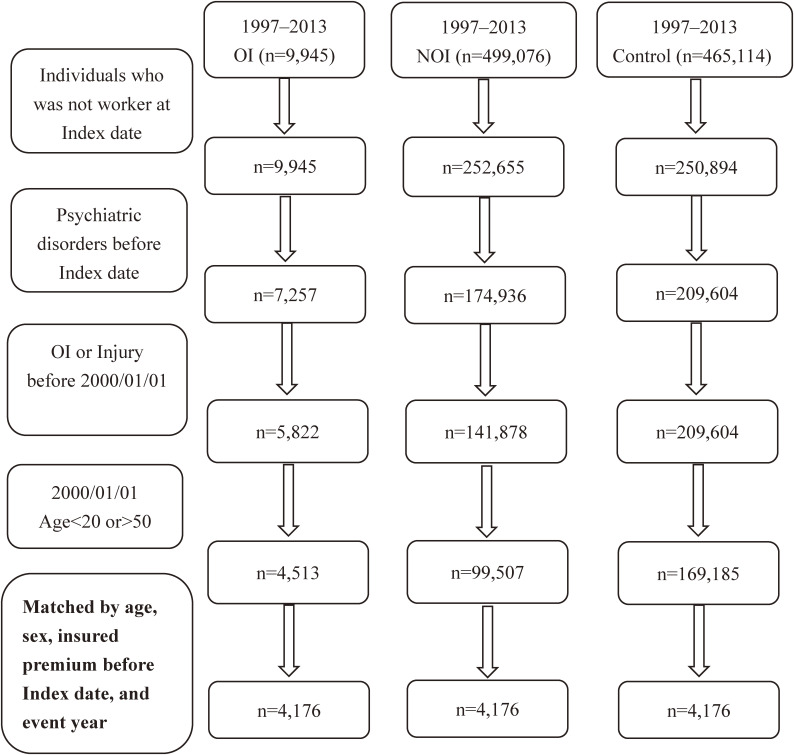
Flow chart showing the procedures of including patients in different study cohorts for final analysis

The procedures of including patients in different study cohorts for final analysis was shown in Figure 1.

### Identification of psychiatric disorders

Participants who had one or more visit codes for either inpatient or outpatient treatment for any listed psychiatric disorder which was diagnosed by psychiatrists between the index date and the end of 2013 were defined as having received treatment for that specific disorder. In each of the following disorders, we identified the date of having received the very first diagnosis of each disorder as the event date.

#### Trauma and stress-related disorders

All subjects in this study were adults and the target injury had occurred in their adulthood. Therefore, we included only the visit code for acute reaction to stress (ICD-9-CM code 308) or adjustment reaction (ICD-9-CM code 309) as treatment for trauma and stress-related disorder (TSRD).^14^

#### Depressive disorders

The following diagnoses were included as depressive disorders: major depressive disorder, single episode (ICD-9-CM code 296.2); major depressive disorder, recurrent episode (ICD-9-CM code 296.3); dysthymic disorder (ICD-9-CM code 300.4); and depressive disorder not elsewhere classified (ICD-9-CM code 311).^15^

#### Anxiety

We defined subjects who had any visit code for the treatment of anxiety states (ICD-9-CM code 300.0), phobic disorders (ICD-9-CM code 300.2), or obsessive-compulsive disorders (ICD-9-CM code 300.3) as having received treatment for anxiety disorders.^16^

#### Alcohol and other substance dependence

In this study, we defined subjects who had received a diagnosis of alcohol dependence syndrome (ICD-9-CM code 303) or drug dependence (ICD-9-CM code 304) as having undergone treatment for alcohol and other substance dependence.^17^

### Statistical analysis

We used descriptive statistical measures to analyze the distribution of the covariates and the incidence rate of psychiatric disorders among subjects. If subjects who met the criteria of having specific psychiatric disorders between the index date and the end of 2013. The date of having received the very first diagnosis of each disorder will be defined as the event date. If subjects who did not received a diagnosis of psychiatric disorders between 2000 and through 2013. Their event date will be the date of withdrawal from the insurance program, death, or the end of 2013. Therefore, the total follow-up period for subjects was the period between index date and event date. We used the cases of having psychiatric disorders divided the total follow-up period of all participants to calculate the incidence rate of psychiatric disorders.

Univariate and multivariate cox proportional hazards regression models were used to calculate hazard ratios (HRs) and 95% confidence intervals (CIs) for psychiatric disorders. Subject who did not received a diagnosis of psychiatric disorders between index date and through 2013, they will be defined as censor. For subjects with censor, their follow-up period will be from the index date to the date of withdrawal from the insurance program, death, or the end of 2013. For subjects with psychiatric disorders, their follow-up period will be from the index date to the first date of having receiving diagnosis of psychiatric disorders. We included all covariates such as age at baseline, sex, insured premium before the index date, hospitalized period, and different population groups into final models to analyze the adjusted relative risks for all variables. We performed all analyses using SAS version 9.4 (SAS Institute Inc., Cary, NC, USA), and we considered a two-sided *P* value of <0.05 as statistically significant.

## RESULTS

Table 1 presents the baseline characteristics of the cohort. After the selection of the NOI and control groups by matching for age, sex, insurance premium at the index date, year of the index date, 4,176 subjects were included in each group. In each group, approximately four-fifth of subjects were men (79.6%), and their average age was 34.0 (standard deviation [SD], 8.6) years. One-quarter of the subjects paid an insurance premium of <NT$20,000, and one-third of the subjects paid an insurance premium of ≥NT$35,000. Most of subjects in OI and NOI groups had less or equal to 7 days of hospitalization immediately after the injury, 65.6% for OI and 97.1% for NOI.

**Table 1.** Characteristic and psychiatric disorders of subjects in different study cohorts

Variables	OI (*n* = 4,176)	NOI (*n* = 4,176)	Control (*n* = 4,176)
**Mean (SD) age at baseline, years**	34.0 (8.6)	34.0 (8.6)	34.0 (8.6)
**Age at baseline, years**			
≤35	2,280 (54.6)	2,280 (54.6)	2,280 (54.6)
>35	1,896 (45.4)	1,896 (45.4)	1,896 (45.4)
**Sex**			
Female	850 (20.4)	850 (20.4)	850 (20.4)
Male	3,326 (79.6)	3,326 (79.6)	3,326 (79.6)
**Insured premium at the Index date**			
<20,000	1,058 (25.3)	1,058 (25.3)	1,058 (25.3)
20,000–24,999	743 (17.8)	743 (17.8)	743 (17.8)
25,000–34,999	990 (23.7)	990 (23.7)	990 (23.7)
35,000–44,999	858 (20.5)	858 (20.5)	858 (20.5)
≥45,000	527 (12.6)	527 (12.6)	527 (12.6)
**Hospitalized period, days**			
≤7	2,741 (65.6)	4,056 (97.13)	4,176 (100.0)
8–14	944 (22.6)	82 (1.9)	—
>15	491 (11.8)	38 (0.9)	—
**Any psychiatric disorders**			
Case	219	196	111
Person year	31,922	32,246	31,138
Incidence rate	6.9	6.1	3.6
**Trauma and stress-related disorder**			
Case	40	36	16
Person year	32,827	32,937	31,579
Incidence rate	1.2	1.1	0.5
**Depressive disorders**			
Case	133	115	69
Person year	32,288	32,611	31,330
Incidence rate	4.1	3.5	2.2
**Anxiety**			
Case	100	102	51
Person year	32,593	32,702	31,472
Incidence rate	3.1	3.1	1.6
**Alcohol and other substance dependence**			
Case	32	41	8
Person year	32,846	32,944	31,649
Incidence rate	1.0	1.2	0.3

SD, standard deviation.

Incidence rate: per 1,000 person-years.

The incidence rates per 1,000 person-years of any psychiatric disorder for the OI, NOI, and control groups were 6.9, 6.1, and 3.6, respectively (Table 1). The incidence rates of any psychiatric disorder, TSRD, depressive disorders, anxiety, and alcohol and other substance dependence were higher in both the OI and NOI groups than in the control group.

The detail prevalence of ICD-9 codes of injury for OI and NOI is shown in eTable 1. The three most common ICD-9-CM codes of injury for OI group were 810–819 fracture of upper limb (30%), 820–829 fracture of lower limb (21.3%), and 880–887 open wound of upper limb (18.9). For NOI group were 920–924 contusion with intact skin surface (26.5%), 880–887 open wound of upper limb (21.9%), and 870–879 open wound of head, neck, and trunk (15.7%).

Table 2 shows the crude HRs for psychiatric disorders. Compared with subjects aged ≤35 years, those aged >35 years had a lower risk of alcohol and other substance dependence. Regarding sex differences, men had a lower risk of depressive disorders and anxiety but a higher risk of alcohol and other substance dependence. Considering the relationship between insurance premium and alcohol and other substance dependence, a dose-response relationship was observed (trend *P* value <0.0005), where those with lower insurance premium had higher risk. Compared with hospitalized days ≤7 days, hospitalized period between 8 and 14 days had higher risk of any psychiatric disorder, depressive disorders, and anxiety, hospitalized period ≥15 days had higher risk of any psychiatric disorder, depressive disorders, and alcohol and other substance dependence. Compared with the control group, the OI and NOI groups had higher risks of any psychiatric disorder, TSRD, depressive disorders, anxiety, and alcohol and other substance dependence.

**Table 2.** Crude hazard ratios of psychiatric disorders in the univariate models using Cox regression

Variable	Any psychiatric disorders	TSRD	Depressive disorders	Anxiety	Alcohol and other substance dependence

	HR (95% CI)				
**Age at baseline, years**					
≤35	1.00	1.00	1.00	1.00	1.00
>35	0.85 (0.72–1.01)	1.02 (0.76–1.38)	0.88 (0.70–1.10)	0.95 (0.74–1.22)	0.63 (0.40–0.99)^*^
**Sex**					
Female	1.00	1.00	1.00	1.00	1.00
Male	0.76 (0.63–0.93)^***^	0.85 (0.59–1.22)	0.73 (0.57–0.94)^*^	0.60 (0.46–0.79)^**^	10.14 (3.20–61.65)^***^
**Insured premium before the Index date**					
<20,000	1.06 (079–1.41)	1.06 (0.50–2.24)	1.21 (0.83–1.76)	0.88 (0.58–1.33)	3.75 (1.32–10.62)^**^
20,000–24,999	1.17 (0.86–1.60)	1.54 (0.72–3.29)	1.23 (0.82–1.85)	0.98 (0.63–1.53)	3.67 (1.25–10.80)^**^
25,000–34,999	0.90 (0.66–1.22)	0.87 (0.40–1.91)	0.97 (0.66–1.44)	0.83 (0.55–1.27)	1.93 (0.64–5.83)
35,000–44,999	0.86 (0.63–1.17)	1.34 (0.64–2.81)	0.77 (0.51–1.18)	0.56 (0.56–1.31)	1.74 (0.56–5.40)
≥45,000	1.00	1.00	1.00	1.00	1.00
**Hospitalized period immediately after the injury, days**					
**≤7**	1.00	1.00	1.00	1.00	1.00
**8**–**14**	1.48 (1.13–1.94)^**^	1.48 (0.79–2.79)	1.69 (1.22–2.35)^**^	1.50 (1.02–2.19)^*^	1.43 (0.71–2.88)
**>15**	1.53 (1.06–2.18)^*^	1.05 (0.38–2.87)	1.86 (1.22–2.86)^**^	1.26 (0.72–2.21)	2.18 (1.00–4.76)^*^
**Group**					
NOI vs Control	1.71 (1.36–2.16)^***^	2.16 (1.22–4.00)^**^	1.60 (1.19–2.16)^**^	1.94 (1.39–2.74)^***^	4.89 (2.42–11.26)^***^
OI vs Control	1.93 (1.53–2.43)^***^	2.41 (1.38–4.43)^**^	1.87 (1.40–2.51)^***^	1.91 (1.37–2.70)^**^	3.83 (1.86–8.93)^**^
OI vs NOI	1.13 (0.93–1.37)	1.11 (0.71–1.75)	1.17 (0.91–1.50)	0.98 (0.75–1.30)	0.78 (0.49–1.24)

CI, confidence interval; HR, hazard ratio; OI, occupational injury; NOI, no occupational injury; TSRD, trauma and stress-related disorder.

^*^P < 0.05; ^**^P < 0.01, ^***^P < 0.0001.

Table 3 presents the adjusted HRs (aHRs) for psychiatric disorders. After adjustments for age, sex, insurance premium, and the days of hospitalization immediately after the injury, the OI and NOI groups had higher risks of any psychiatric disorder, TSRD, depressive disorders, anxiety, and alcohol and other substance dependence than did the control group.

**Table 3.** Adjusted hazard ratio of psychiatric disorders in the multivariable models using Cox regression

Variable	Any psychiatric disorders	TSRD	Depressive disorders	Anxiety	Alcohol and other substance dependence

	aHR (95% CI)				
**Age at baseline, years**					
≤35	1.00	1.00	1.00	1.00	1.00
>35	0.88 (0.74–1.05)	0.66 (0.43–1.02)	0.93 (0.74–1.16)	0.96 (0.75–1.24)	0.73 (0.46–1.16)
**Sex**					
Female	1.00	1.00	1.00	1.00	1.00
Male	0.76 (0.62–0.93)^**^	0.75 (0.47–1.21)	0.73 (0.57–0.94)^*^	0.59 (0.45–0.78)^**^	10.96 (2.69–44.62)^***^
**Insured premium before the Index date**					
<20,000	0.99 (0.74–1.32)	0.96 (0.45–2.03)	1.13 (0.77–1.65)	0.79 (0.52–1.20)	4.09 (1.44–11.61)^**^
20,000–24,999	1.11 (0.82–1.52)	1.42 (0.66–3.05)	1.16 (0.77–1.75)	0.91 (0.58–1.42)	3.89 (1.32–11.47)^*^
25,000–34,999	0.86 (0.64–1.17)	0.84 (0.38–1.84)	0.93 (0.62–1.37)	0.77 (0.50–1.17)	2.17 (0.72–6.55)
35,000–44,999	0.86 (0.63–1.17)	1.39 (0.66–2.94)	0.77 (0.51–1.18)	0.83 (0.54–1.28)	1.95 (0.63–6.06)
≥45,000	1.00	1.00	1.00	1.00	1.00
**Hospitalized period immediately after the injury, days**					
**≤7**	1.00	1.00	1.00	1.00	1.00
**8**–**14**	1.21 (0.90–1.62)	1.12 (0.56–2.22)	1.45 (1.00–2.09)^*^	1.30 (0.85–1.98)	1.23 (0.58–2.66)
**>15**	1.27 (0.87–1.86)	0.83 (0.29–2.34)	1.62 (1.03–2.57)^*^	1.14 (0.63–2.05)	1.78 (0.77–4.10)
**Group**					
NOI vs Control	1.70 (1.34–2.14)^***^	2.16 (1.20–3.89)^*^	1.57 (1.16–2.11)^**^	1.93 (1.38–2.70)^**^	4.73 (2.22–10.10)^***^
OI vs Control	1.78 (1.39–2.29)^***^	2.39 (1.29–4.43)^**^	1.58 (1.14–2.19)^***^	1.77 (1.22–2.55)^**^	3.26 (1.43–7.43)^**^
OI vs NOI	1.05 (0.85–1.30)	1.11 (0.68–1.81)	1.01 (0.76–1.34)	0.92 (0.68–1.24)	0.69 (0.41–1.16)

aHR, hazard ratio, adjusted for age at base line, sex, insured premium before the index date, hospitalized period (days), and group; CI, confidence interval; OI, occupational injury; NOI, no occupational injury; TSRD, trauma and stress-related disorder.

^*^P < 0.05; ^**^P < 0.01, ^***^P < 0.001.

Considering the impact of traumatic brain injury (TBI) on mental conditions, we also divided OI and NOI groups into OI with or without TBI and NOI with or without TBI, and then compared the HR of psychiatric disorders of each subgroup with control group. The crude and adjusted HR for psychiatric disorders are shown in eTable 2 and eTable 3. After adjustments for age, sex, insurance premium, and the days of hospitalization immediately after the injury, the OI with or without TBI and NOI with or without had higher risks of any psychiatric disorder, depressive disorders, and alcohol and other substance dependence than did the control group.

**Figure 2.**
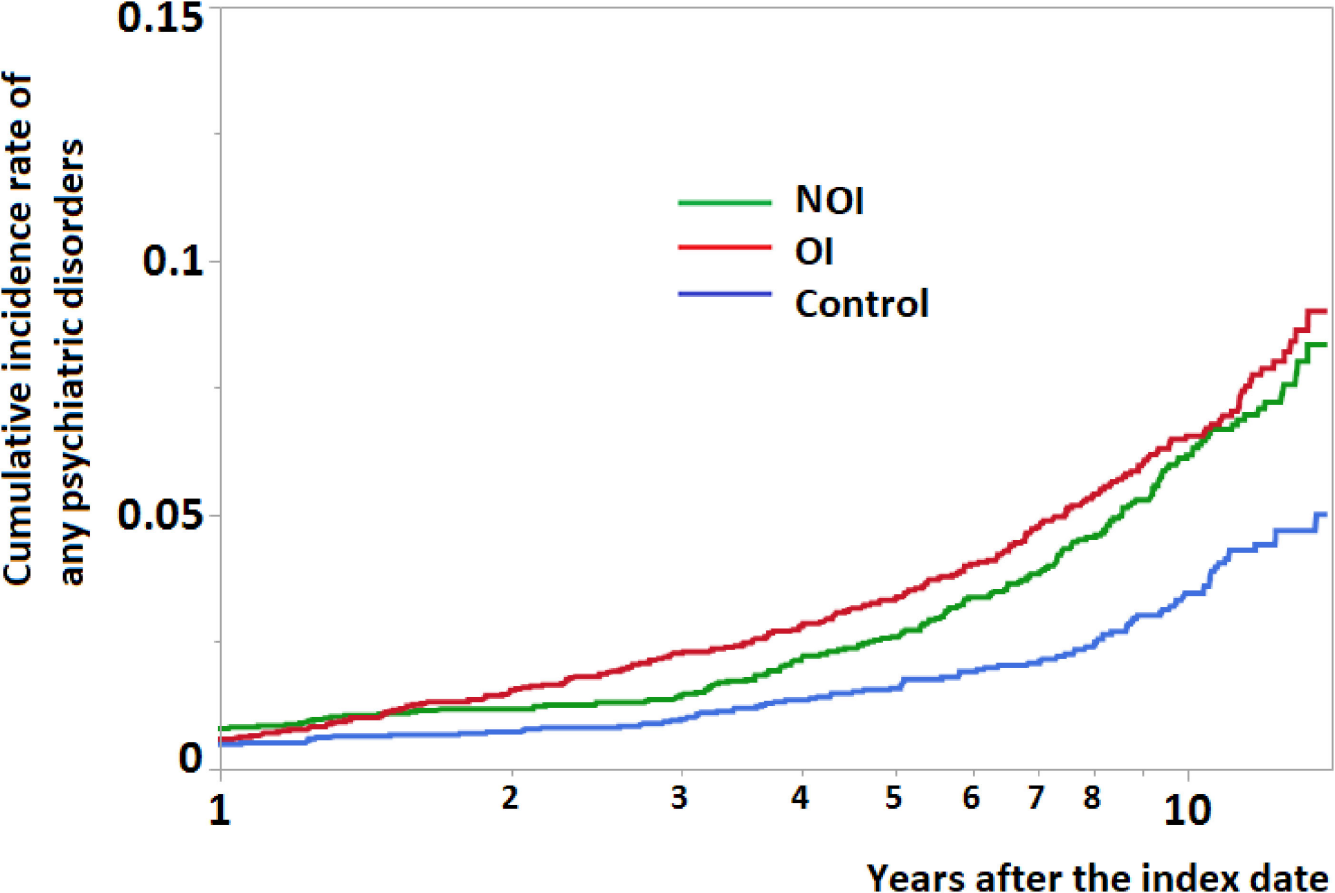
Cumulative incidence rate of any psychiatric disorders in different study cohorts

Figure 2 displays the cumulative incidence rate of any psychiatric disorders in different study cohorts. In the beginning after the injury, the OI and NOI groups only had slightly higher cumulative incidence rate of any psychiatric disorders than control group. However, as time went by, comparing with control group, the OI and NOI groups had significantly higher cumulative incidence rate of any psychiatric disorders up to 10 years after injury.

## DISCUSSION

This is the first study to assess the long-term psychiatric consequences of OI. The incidence rates of psychiatric disorders, namely TSRD, depressive disorders, anxiety, and alcohol and other substance dependence, were higher in subjects with OI than in those without injury. The high incidence rate observed in subjects with OI was similar to that observed in those with NOI. In addition, clinic and emergency visits due to psychiatric disorders were higher in the OI group than in the control group up to 10 years after injury.

Despite that higher than 99% of population is covered by the national insurance, a certain percentage of people does not use the insurance. For example, in this database, approximately 7.4% of people were insured, but did not have relevant medical records. It is known that lack of usage in health insurance was caused by those who only used private insurance, who could not afford even deductible fees, or who resided most of their time abroad. Using these people as controls might underestimate the occurrence of psychiatric conditions among those without injuries. We therefore included those who ever used the most common condition, upper respiratory infection, as a criteria of ever using health insurance. These provided a better representation of the population who use the National Health Insurance.

A study reported an increased occurrence of psychiatric disorders after injury.^18^ Individuals exposed to a threat of death, severe degree of injury, or threat to physical integrity may develop psychological reactions to the trauma, including fear-based, dysphoric and anhedonic, aggressive/externalizing, and guilt/shame symptoms.^19^ These reactions may lead to the further development of psychiatric disorders.^20^ Zatzick and coworkers screened for mental conditions in 2,707 injury victims requiring surgical inpatient treatments from 69 American hospitals at 12 months after injury; more than 20% of the patients had PTSD and/or depression.^21^ In Australia, Bryant et al found that among 1,084 injured patients, 23% had psychiatric disorders at 12 months after injury, with depression and anxiety disorder being the most common newly diagnosed conditions.^18^ Studies involving longer follow-up periods have revealed that a significant proportion of injury victims had psychiatric disorders,^6^^,^^10^^,^^22^ including major depression, PTSD, substance use disorder, and generalized anxiety disorder. However, long-term comparisons of the occurrence of psychiatric disorders between injury victims and uninjured people are scant. The results of the current study reveal a high incidence of psychiatric disorders in subjects with OI than in those without injury matched by age, sex, insurance premium at the index date, and year of the index date. The observed long-term increase in the occurrence of psychiatric disorders not only confirms previous findings but also provides crucial evidence that the increased occurrence of psychiatric conditions continued and required medical attention among subjects with OI many years after the injury event.

The observed HRs between the OI and NOI groups did not differ statistically for TSRD, depressive disorders, anxiety, and alcohol and other substance dependence. Individuals encountered traumatic injury may experience medical procedures, chronic physical pain, occupational disruption, financial hardship, protracted adversarial compensation process.^23^^,^^24^ These costly and subsequently life-altering situations have been attributed to the stressors that occur in aftermath of the injury.^18^ As a result, some individuals may response to these stressors is expected to include some emotional reaction.^25^ A slightly increased of HR for depressive disorders was observed in the OI group relative to the NOI group. The exact cause of this observation remains unclear. However, previous studies have suggested several causes of increased stress and depression among workers, including the following: working with a compensation system toward conceived fairness in workers’ rights^26^ and the associated stress and trouble,^27^ prolonged musculoskeletal symptoms resulting from OI,^28^ unstable employment, and difficulties in return to work.^10^ Despite the uncertainty of the exact cause, the higher occurrence of depressive disorders in the OI group than in the NOI group implies a suboptimal postinjury compensation and psychological care system. Strategies and policy to improve post-OI care are warranted to minimize the occurrence of psychiatric disorders after OI.

The International Labor Organization (ILO) added mental and behavior-related diseases to the list of occupational diseases in 2010.^29^ However, not all countries adopted the ILO list. The Report on the Current Situation in Relation to Occupational Diseases’ Systems in European Union (EU) Member States and European Free Trade Association/European Economic Area Countries^30^ documented that many EU member states have recognized and compensated PTSD resulting from work-related accidents. However, other psychiatric disorders were not included. Therefore, when an OI victim develops psychiatric disorders such as depression several months after OI, this psychiatric condition may be considered occupation unrelated. In the United States, the Occupational Injury and Illness Classification Manual (OIICM) states that when a mental disorder is secondary to a traumatic incident, it will be recognized as an occupational illness.^31^ However, workers’ compensation benefits vary by states.^32^ Moreover, without the knowledge that psychiatric disorders can occur many years after OI, a mental disorder may not be recognized as related to a traumatic event.

In 2016, the global burden of disease for OI was estimated to be a 22 million reduction in DALYs, accounting for 2.0% of all diseases.^1^ However, the DALYs of mental disorders resulting from OI were not included in the estimation. The consideration of environmental and occupational causes, including psychiatric sequels, may provide a more comprehensive picture of the contribution of OI to DALY loss.

Previous studies have reported that patients with traumatic brain injury (TBI) had higher risk of developing psychiatric disorders after the traumatic injury.^33^^,^^34^ However, in this study, we did not find that injured individuals with TBI had increased risk of having psychiatric disorders compared for those without TBI, these results are shown in eTable 2 and eTable 3. Consistent with previous findings,^35^^–^^38^ our results show a significant sex difference in several psychiatric conditions. Women were more likely to have anxiety and depressive disorders. By contrast, men were 8.7 times more likely to have alcohol-related disorders. These differences result from biological, psychosociocultural, and environmental factors.^37^^,^^39^ Severe injuries were associated with greater length of hospital stay.^40^ In this study, we found that compared with subjects had less or equal to 7 days of hospitalization after injury, those with longer length of hospital stay had higher risk for developing depressive disorders. This result was similar with previous studies. The longer length of hospital stay has been reported as a crucial factor for injured workers’ mental health.^3^^,^^10^ Workers with longer length of hospital stay after injury would have higher scores of psychological symptoms even years after the injury.^3^^,^^10^ Consistent with the findings of previous surveys,^41^^–^^44^ in this study, the risk of alcohol and other substance dependence was higher in subjects who paid a lower insurance premium, which is a proxy of socioeconomic status.^45^ These discrepancies in the risk of psychiatric disorders provide information that can assist in designing preventive strategies against psychiatric conditions after OI.

The aim of this investigation was to determine whether occupational injury result in increased psychiatric diseases. From our findings, psychiatric conditions were increased with both OI and NOI. This is compatible with previous studies.^5^^,^^10^^,^^46^^,^^47^ However, it is uncertain whether increased psychiatric disorders were caused by any injury, or specifically injury by occupational factors. In other words, are there factors involved in workplace that added to the increased psychiatric disorders purely caused by injury? From the comparisons between psychiatric conditions from OI and NOI, we did not find additional increment of psychiatric conditions among workers injured by workplace insults, as compared to workers injured by non-workplace ones.

This study has several strengths. First, the study population was a nationally representative sample of the Taiwanese population insured under the NHI. The NHI program’s universal coverage of more than 99% of all residents in Taiwan enabled ready access to health services, regardless of socioeconomic status and/or residential location. Second, the large sample size allowed for careful matching for age, sex, insurance premium, and year of the index date, thus minimizing potential factors contributing to psychiatric diseases. Third, the retrospective cohort study design ensured the temporal sequence of injury and mental disorders. Fourth, the outcome of psychiatric diseases was based on the diagnosis made by psychiatrists and was thus less likely to be biased. This study has some limitations. First, this study did not include risk factors for psychiatric disorders, such as marital status, education level, family history of psychiatric disorders, occupational type, work pattern, and the outcome of injury. Potential biases could have been introduced if these risk factors were also linked to the risk of OI. However, the increase in the injury rate in those with psychiatric conditions has been reported to be only 28–60%.^48^^,^^49^ Second, one limitation of the health insurance data is the lacking of individually assessed severity of injury. Hospitalization period was thus used as a surrogate for severity. In Taiwan, higher than 99% of the people were insured and thus treated for injuries. In the meantime, unnecessarily prolonged hospitalization was scrutinized by the relevant authority in a standardized manner. Therefore, duration of hospitalization is a reasonable indicator of severity. This is supported by our findings that hospital period is highly related to occurrence of psychiatric disorders. Third, psychiatric disorders are underdiagnosed and undertreated in Taiwan.^50^^,^^51^ This likely also applied to our study population. However, the Taiwan NHI program covers more than 99.0% of the population.^12^ Thus, it is unlikely that injured people were less underdiagnosed compared with uninjured people.

### Conclusion

The results of this long-term follow-up study show an increased occurrence of psychiatric disorders after OI and NOI, especially, TSRD and depressive disorders. Increased needs for medical facilities lasted for at least 10 years after OI and NOI. A more thorough psychiatric evaluation and longer follow-up period for psychiatric conditions are warranted after OI and NOI.

## APPENDIX A. SUPPLEMENTARY DATA

eTable 1. The prevalence of different International Classification of Disease (ICD)-9 code of injury (ICD-9-CM codes 800.xx to 999.xx)

eTable 2. Crude hazard ratios of psychiatric disorders in the univariate models using Cox regression

eTable 3. Adjusted hazard ratio of psychiatric disorders in the multivariable models using Cox regression
