# Supplementary material for: Occupational and Non-occupational Injuries Can Result in Prolonged Augmentation of Psychiatric Disorders
Source: J Epidemiol. 2022 Jan 5;32(1):12–20. doi: 10.2188/jea.JE20200374 (PMC8666318; doi:10.2188/jea.JE20200374)
Supplement: Supplementary file 1 [file je-32-012-s001.pdf]

e Table 1. The prevalence of different International Classification of Disease (ICD)-9 code of injury (ICD-9-CM codes 800.xx to 999.xx)

ICD 9 code	OI	Non OI
	n (%)	n (%)
800-804 Fracture of Skull	158 (3.9)	61 (1.5)
805-809 Fracture of Spine and Trunk	311 (8.1)	62 (1.5)
810-819 Fracture of Upper Limb	1,162 (30.4)	220 (5.3)
820-829 Fracture of Lower Limb	814 (21.3)	150 (3.6)
830-839 Dislocation	174 (4.6)	67 (1.6)
840-848 Sprains and Strains of Joints and Adjacent Muscles	174 (4.6)	261 (6.3)
850-854 Intracranial Injury, Excluding Those with Skull Fracture	380 (10.0)	319 (7.6)
860-869 Internal Injury of Chest, Abdomen, and Pelvis	129 (3.4)	33 (0.8)
870-879 Open Wound of Head, Neck, and Trunk	354 (9.3)	656 (15.7)
880-887 Open Wound of Upper Limb	723 (18.9)	913 (21.9)
890-897 Open Wound of Lower Limb	231 (6.0)	439 (10.5)
900-904 Injury to Blood Vessels	68 (1.8)	17 (0.4)
905-909 Late Effects of Injuries, Poisonings, Toxic Effects, and other External Causes	93 (2.4)	12 (0.3)
910-919 Superficial Injury	300 (7.9)	667 (16.0)
920-924 Contusion with Intact Skin Surface	403 (10.6)	1,105 (26.5)
925-929 Crushing Injury	394 (10.3)	120 (2.9)
930-939 Effects of Foreign Body Entering through Orifice	4 (0.1)	128 (3.1)
940-949 Burns	198 (5.2)	133 (3.2)
950-957 Injury to Nerves and Spinal Cord	150 (3.9)	23 (0.6)
958-959 Certain Traumatic Complications and Unspecified Injuries	168 (4.4)	142 (3.4)
960-979 Poisoning by Drugs, Medicinals and Biological Substances	1 (0.0)	16 (0.4)
980-989 Toxic Effects of Substances Chiefly Nonmedicinal as To Source	7 (0.2)	38 (0.9)
990-995 Other and Unspecified Effects of External Causes	5 (0.1)	100 (2.4)
996-999 Complications of Surgical and Medical Care, Not Elsewhere Classified	47 (1.2)	18 (0.4)

eTable 2. Crude hazard ratios of psychiatric disorders in the univariate models using Cox regression

Variable	Any psychiatric disorders	TSRD	Depressive disorders	Anxiety	Alcohol and other substance dependence
			**HR (95% CI)**		
**Group**					
NOI with TBI vs. Control	1.88 (1.18- 3.00)**	1.85 (0.54- 6.33)	2.16 (1.24- 3.78)*	2.29 (1.23- 4.31)**	6.16 (2.02- 18.83)**
NOI with no TBI vs. Control	1.69 (1.33- 2.15)***	2.20 (1.21- 3.99)**	1.54 (1.13- 2.09)**	1.90 (1.35- 2.68)**	4.76 (2.21- 10.23)***
OI with TBI vs. Control	2.32 (1.56- 3.43)**	1.96 (0.66- 5.86)	2.54 (1.57- 4.10)**	1.23 (0.58- 2.59)	7.84 (2.94- 20.88)***
OI with no TBI vs. Control	1.88 (1.48- 2.37)***	2.47 (1.37- 4.45)**	1.77 (1.31- 2.40)**	2.01 (1.43- 2.83)***	3.27 (1.47- 7.28)**

CI, confidence interval; HR, hazard ratio; TSRD, trauma and stress-related disorder.

*p<0.05; ***P*<0.01, ***p<0.0001

＊Traumatic Brain Injury: ICD 9 code: 800-804 Fracture of Skull or 850-854 Intracranial Injury, Excluding Those with Skull Fracture

eTable 3. Adjusted hazard ratio of psychiatric disorders in the multivariable models using Cox regression

Variable	Any psychiatric disorders	TSRD	Depressive disorders	Anxiety	Alcohol and other substance dependence
	aHR (95% CI)				
**Group**					
NOI with TBI vs. Control	1.83 (1.14- 2.91)*	1.87 (0.54- 6.42)	2.02 (1.15- 3.54)*	2.22 (1.18- 4.17)*	5.94 (1.94- 18.26)**
NOI with no TBI vs. Control	1.68 (1.33- 2.13)***	2.19 (1.20- 3.97)*	1.52 (1.12- 2.06)**	1.90 (1.34- 2.67)**	4.62 (2.14- 9.93)***
OI with TBI vs. Control	2.06 (1.36- 3.10)**	1.88 (0.61- 5.81)	2.04 (1.23- 3.39)**	1.06 (0.49- 2.28)	7.44 (2.65- 20.89)**
OI with no TBI vs. Control	1.75 (1.36- 2.26)***	2.45 (1.31- 4.59)**	1.53 (1.10- 2.13)*	1.87 (1.29- 2.71)**	2.82 (1.22- 6.55)*

aHR, hazard ratio mutually adjusted for age at base line, sex, insured premium before the index date, hospitalized period; CI, confidence interval; TSRD, trauma and stress-related disorder.

*p<0.05; ***P*<0.01, ***p<0.0001

＊Traumatic Brain Injury: ICD 9 code: 800-804 Fracture of Skull or 850-854 Intracranial Injury, Excluding Those with Skull Fracture
